# Characterization and 454 pyrosequencing of Major Histocompatibility Complex class I genes in the great tit reveal complexity in a passerine system

**DOI:** 10.1186/1471-2148-12-68

**Published:** 2012-05-15

**Authors:** Irem Sepil, Hooman K Moghadam, Elise Huchard, Ben C Sheldon

**Affiliations:** 1 Department of Zoology, Edward Grey Institute, University of Oxford, South Parks Road, Oxford, OX1 3PS, UK; 2 Behavioral Ecology and Sociobiology Unit, German Primate Centre, Kellnerweg 4, Göttingen, 37077, Germany; 3 CRC ‘Evolution of Social Behavior’, Georg-August University, Kellnerweg 6, Göttingen, 37077, Germany

## Abstract

**Background:**

The critical role of Major Histocompatibility Complex (*Mhc*) genes in disease resistance and their highly polymorphic nature make them exceptional candidates for studies investigating genetic effects on survival, mate choice and conservation*.* Species that harbor many *Mhc* loci and high allelic diversity are particularly intriguing as they are potentially under strong selection and studies of such species provide valuable information as to the mechanisms maintaining *Mhc* diversity. However comprehensive genotyping of complex multilocus systems has been a major challenge to date with the result that little is known about the consequences of this complexity in terms of fitness effects and disease resistance.

**Results:**

In this study, we genotyped the *Mhc* class I exon 3 of the great tit (*Parus major*) from two nest-box breeding populations near Oxford, UK that have been monitored for decades. Characterization of *Mhc* class I exon 3 was adopted and bidirectional sequencing was carried using the 454 sequencing platform. Full analysis of sequences through a stepwise variant validation procedure allowed reliable typing of more than 800 great tits based on 214,357 reads; from duplicates we estimated the repeatability of typing as 0.94. A total of 862 alleles were detected, and the presence of at least 16 functional loci was shown - the highest number characterized in a wild bird species. Finally, the functional alleles were grouped into 17 supertypes based on their antigen binding affinities.

**Conclusions:**

We found extreme complexity at the *Mhc* class I of the great tit both in terms of allelic diversity and gene number. The presence of many functional loci was shown, together with a pseudogene family and putatively non-functional alleles; there was clear evidence that functional alleles were under strong balancing selection. This study is the first step towards an in-depth analysis of this gene complex in this species, which will help understanding how parasite-mediated and sexual selection shape and maintain host genetic variation in nature. We believe that study systems like ours can make important contributions to the field of evolutionary biology and emphasize the necessity of integrating long-term field-based studies with detailed genetic analysis to unravel complex evolutionary processes.

## Background

Genes of the *Mhc* encode cell-surface proteins responsible for the recognition and presentation of foreign antigens to T-lymphocytes, which then initiate an immune response against pathogens [1]. The *Mhc* is known to be the most variable gene group in vertebrates, both in terms of allelic diversity and gene number [2]. Numerous studies have suggested that selection from parasites is the major factor driving this polymorphism, whereas the role of sexual selection has also been demonstrated in several species [3-5]. Interest in understanding *Mhc* genes is growing significantly as more studies demonstrate implications of their diversity in the context of survival [6], mate choice [7], conservation [8] and even speciation [9]. The number of *Mhc* genes can differ greatly between and within species, and may show no sequence orthology between genera or orders – e.g. class I gene number and organization differences between human and chimpanzees (reviewed in [10]). Differences in the number and organization of *Mhc* genes are explained by a birth and death model of evolution [11]. According to this model, new genes are formed by duplication events and, while some retain their function in the genome, others are inactivated or deleted [12]. Because an individual *Mhc* molecule can recognize and bind to a few antigens only, which is determined by the amino acid composition in their antigen-binding site (ABS), gene duplications and polymorphism at the ABS are thought to be adaptations enabling individuals to respond to a greater variety of antigens [13].

The mammalian *Mhc* is a gene-dense region that is divided into subfamilies with similar function. Class I and class II genes are considered ‘classical’ *Mhc* genes to indicate their polymorphic, highly expressed, structure; and antigen processing and presenting function. In sharp contrast to mammals with multiple class I and II loci, the avian model organism, the chicken has a “minimal essential *Mhc*”, a term referring to its surprisingly small, densely packed *Mhc* region with only two class I and II genes [14,15]. However, recent studies have shown that “minimal essential *Mhc*” does not hold for other avian orders, as a larger number of class I and II genes have been detected in passerines [16]. The recent in-depth characterization of zebra finch *Mhc* demonstrated that more than ten *Mhc* class I and class II loci are located on different chromosomes in this species ([17], but see [18]). Many of the genes were suspected to be pseudogenes and only one *Mhc* class I locus appeared to be transcribed, contrasting to the pattern observed in other passerine species, where many functional class I loci have been described [19-22]. Similarly the recent characterization of *Mhc* class II loci exon 2 in the collared flycatcher revealed the presence of at least nine expressed (and a larger number of pseudogene) loci, confirming the complexity and variable evolutionary dynamics of this region in Passeriformes [23].

The presence of multiple loci and high allelic diversity in passerines make them intriguing species for *Mhc* studies as they are potentially under strong selection, and thus can provide valuable information on the mechanisms maintaining *Mhc* polymorphism. However the complexity of multilocus *Mhc* genes also poses a great challenge for accurate genotyping. In species where the *Mhc* has undergone rapid duplication, gene conversion and recombination, the genes are often tightly linked to each other and alleles are shared among loci, making it almost impossible to identify and isolate independent loci. Therefore simultaneous amplification of multiple loci becomes necessary in such systems [24].

It is surprising that until recently the importance of accurate genotyping has been somewhat neglected in species harbouring complex multilocus *Mhc* systems [25]. Initial characterization of *Mhc* genes is essential to fully understand the architecture of the system and to design primers that would potentially amplify all the alleles of interest, so that precise genotyping could be achieved [26]. Moreover the fact that some alleles might be non-functional further complicates the situation and obligates the differentiation of functional alleles from the non-functional ones through historical selection tests and screening of cDNA libraries. The method chosen for *Mhc* genotyping is also of great importance. There are a few indirect genotyping techniques that have been widely used to date; although these methods require extensive initial optimization, often necessitate further characterization via cloning and sequencing, and are still insufficient when genotyping complex multilocus systems (reviewed in [27,28]). The application of Next Generation Sequencing (NGS) methods for *Mhc* screening has recently been introduced as a promising alternative to the conservative typing methods and has the potential to provide high resolution, accurate and large-scale genotyping, thus solving three problems that have plagued previous studies in this field [24,28,29]. Especially in projects where sample sizes are high, 454-pyrosequencing may outperform the other methods both in terms of the time it takes and per sample cost. One major drawback of the application of high-throughput NGS is its error-prone sequencing technology; therefore strict quality control is crucial to distinguish true alleles from sequencing artefacts (reviewed in [25]). To date relatively few published studies have used 454-pyrosequencing method for *Mhc* genotyping in non-model vertebrates; all succeeded in identifying real and artificial sequences. The repeatability of the results was confirmed for all but one, by cloning or running a fraction of the samples in duplicates [24,28,30]. In one study, that by Zagalska-Neubauer et al. [23], the repeatability of the duplicate samples was low, because the coverage of reads was not sufficient for reliable genotyping. However this did not prevent full characterization of diversity at species level.

These recent studies demonstrate that 454 or similar technologies can be employed for accurate typing of complex *Mhc* loci. Reliable genotyping of multilocus *Mhc* alleles is, however, still not sufficient for determining what equates to functionally important alleles. *Mhc* alleles commonly share the same ABS aminoacids and have similar antigen-binding motifs; hence their allelic differences are not always functionally relevant (discussed in [26]). Therefore a growing body of evidence across taxa highlights the importance of clustering *Mhc* alleles into functional supertypes and considering these supertypes as the unit of selection [31-34]. For instance Trachtenberg et al. [35] showed that rare human leukocyte antigen (HLA) supertypes confer a strong advantage in responding to HIV infection, independent of the contribution of single alleles.

Here, we describe an approach for characterizing and genotyping *Mhc* class I loci exon 3 in natural great tit (*Parus major*) populations. Genes of *Mhc* class I have been shown to influence avian malaria resistance [36-39], female mate choice [40] and extra-pair paternity [41,42] in other passerine species. Initially we undertook a comprehensive characterization effort to detect the diversity of allelic variants at exon 3, and used 454 pyrosequencing for genotyping. Using this technique we genotyped a fragment of *Mhc* class I exon 3 of several hundred great tits simultaneously; and applied a stepwise variant validation procedure to separate true alleles (the procedure was based on the methods defined by Zagalska-Neubauer et al. [23] and Galan et al. [28]). Then we differentiated the functional alleles from the non-functional ones, first by investigating the presence of stop codons in sequences and examining the phylogenetic relationship between allelic clusters, and secondly through historical selection tests. Finally we clustered the *Mhc* alleles into functional supertypes, to determine the biologically meaningful *Mhc* variants of each individual. Our main aim was to describe an approach for accurate *Mhc* characterization and typing, a crucial prerequisite for studies investigating the role and importance of complex *Mhc* loci in non-model vertebrates.

## Methods

### Samples and DNA extractions

We analyzed 1496 first year or adult great tits, sampled between 2006–2010 in Wytham Woods and Bagley Woods nest-box breeding populations. Both populations have been monitored continuously since the 1960s and 1990s respectively, and are located within a few km of Oxford, UK. All great tits were ringed with aluminium bands for individual recognition. Blood was collected by wing or jugular venipuncture under UK Home Office licence (PPL 30/2409), and stored in SET Buffer at −80°C until DNA extraction. Total genomic DNA was extracted using standard ammonium acetate method and stored in AE Buffer (Qiagen). DNA concentration was measured using a Picogreen assay (Quant-iT Picogreen dsDNA Assay Kit, Invitrogen) and samples were diluted to 5-20 ng/μl when necessary.

### Development of primers

**Figure 1:**
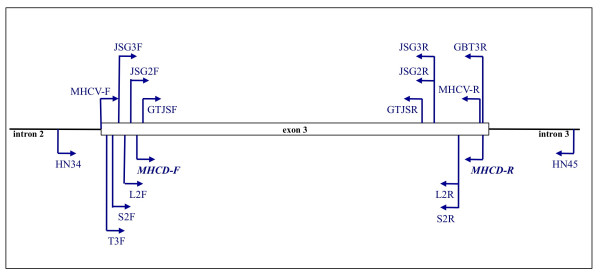
Schematic overview of the location of primers used in the study.

We used degenerate primers HN34 and HN45 [43], as well as primers T3F and GTJSF in the 3′ direction and GBT3R and GTJSR in the 5′ direction, identified from the alignment of tit sequences available in NCBI (great tits [GenBank: AF346821– AF346832], green-backed tits [GenBank: EF446972–EF446988] and blue tits [GenBank: AM232705– AM232717]) to isolate complete and partial exon 3 sequences. Then we designed specific primers within this exon using the consensus from these sequences (see Figure 1 and Table 1 for the sequence and location of each primer). *Mhc* class I exon 3 was chosen as the target region, as it bears most of the ABS. PCR reactions were run using Platinum® Taq DNA Polymerase Kit (Invitrogen). PCR amplification was performed in 25 μl reactions, with the following final concentrations: 1x PCR Buffer, 1.5 mM MgCl_2_, 0.5 μM of each primer (forward and reverse), 0.5 units of Platinum Taq Polymerase (Invitrogen) and 0.125 mM of each dNTP. 2 μl of extracted DNA (5-20 ng/μl) was added to this mix. The reaction was run for 30 cycles at 95°C for 45 s, 57°C-65°C for 45 s and 72°C for 45 s on a GeneAmp PCR System 9700 thermal cycler (Applied Biosystems). PCR products were purified using MinElute 96 UF PCR Purification Kit (Qiagen), ligated in plasmid vector and transformed to bacteria using pCR8/GW/TOPO TA Cloning Kit (Invitrogen). Each primer pair was tested on four individuals – two males (first year and adult) and two females (first year and adult) that were sampled in different areas of the population. Twelve clones from each individual were randomly selected and reamplified using the same PCR conditions. The amplicons were sequenced directly by dye terminator cycle sequencing (BigDye version 3.1) and loaded on an ABI PRISM 310 Automated Sequencer (Applied Biosystems). The sequences were edited in Sequencher version 4.2 (GeneCode) [44] and aligned using BioEdit Sequence Alignment Editor [45].

**Table 1:** **Primers used for amplifying *Mhc *class I exon 3 in great tits**

**Primer**	**Sequence (5′ - > 3′)**	**PCR**	**Reference**
HN34	CCATGGGTCTCTGTGGGTA	gDNA	[39]
HN45	CCATGGAATTCCCACAGGAA	gDNA	[39]
T3F	TCCACACCATACAGCGAGTT	gDNA, cDNA	present study
GBT3R	TTTACGCTCCAGCTCTTTCC	gDNA, cDNA	present study
GTJSF	CGGCTGTGACCTCCTGTCC	gDNA	present study
GTJSR	ATTCYGGGCAGATGTGCTT	gDNA	present study
S2F	CACACCTCACAGTGGCTTTA	gDNA, cDNA	present study
S2R	CAGCTCTTTCTGCCCATATC	gDNA, cDNA	present study
L2F	CAATGGCTTTATGGCTGTGAC	gDNA, cDNA	present study
L2R	CCAGCTCTTTCCTCCCGTAT	gDNA, cDNA	present study
JSG2F	AGTTTCCGGCTGTGACCTC	gDNA	present study
JSG2R	GCCCGTATTCGACGTATTTC	gDNA	present study
JSG3F	CATACAGTGGCTTTATGGCTGT	gDNA	present study
JSG3R	CCCGTATCCGGTGTATTTCC	gDNA	present study
MHCV-F	CCCAGGTCTCCACACCATAC	gDNA	present study
MHCV-R	AGCTCTTTCCGCCCGTATT	gDNA	present study
MHCD-F	TTMYGGCTGTGACCTCCTG	gDNA, cDNA	present study
MHCD-R	TTGCGCTYCAGCTCTTTC	gDNA, cDNA	present study

The primers were developed in the order from top to bottom (with the exception of HN34-45). MHCD-F and MHCD-R are the final primers designed for 454 pyrosequencing. F indicates forward and R indicates reverse primers. gDNA indicates amplification of genomic DNA and cDNA indicates amplification of cDNA libraries with the specified primer.

After isolating a large number of variable *Mhc* class I sequences, we used the consensus from the obtained sequences to design a degenerate primer pair (MHCD-F and MHCD-R) that would potentially amplify all the expressed alleles that are present in the great tit. By using these degenerate primers we aimed to minimize the risk of missing out a fraction of allelic variation that might bias the results of the studies to follow. The primers amplified a 212–221 basepair fragment (without primers) of *Mhc* class I exon 3 (78%) in the great tit. Four individuals were amplified with the primers under the same PCR conditions and 65°C for annealing temperature. The PCR products were cleaned, cloned and sequenced as described above.

### RNA extraction, cDNA synthesis, RT PCR

Total RNA was extracted from four great tit blood samples for expression analysis. Blood samples collected via jugular venipuncture were immediately placed into RNAprotect Animal Blood Tubes (Qiagen) for RNA stabilization and were stored at 4°C overnight. RNA isolation was done using RNeasy Protect Animal Blood Kit (Qiagen) and reverse transcription was carried out with Omniscript Reverse Transcription Kit (Qiagen). First strand cDNA was synthesized in a final volume of 20 μl with the following conditions: 1x Buffer RT, 1 μM of Oligo-dT primer, 10 units of RNase inhibitor, 4 units of Omniscript Reverse Transcriptase, 10.5 mM of each dNTP and 1 μl of template RNA. The mixture was incubated for 60 min at 37°C and a 2 μl aliquot of the finished reverse-transcription reaction was used for Two-Tube RT-PCR. We used four different primer pairs for RT-PCR (Table 1) and adjusted the same PCR conditions that we described for genomic DNA amplification. Again the PCR products were cleaned, cloned and sequenced.

### Tagged primer design; amplicon preparation, dilution and pooling

454 pyrosequencing was performed on 1532 genomic DNA samples from 1492 great tits, collected between 2006–2010 in Wytham and Bagley Woods; and cDNA libraries of four Wytham Wood great tits. The individuals with cDNA libraries were not included in the genomic DNA sequencing panel, since the RNA stabilization step prevented DNA isolation from these samples. Forty genomic DNA samples were randomly chosen and ran in duplicates to estimate the repeatability of the results. In order to maximize the throughput for 1536 great tit samples from the bidirectional sequencing run, we used the 16 regions of the Pico Titer Plate individually (the maximum physical separation currently available). Therefore the samples were divided into 16 sets of 96 individuals and individuals in each region were differentiated by the use of multiplex identifiers (MID).

MHCD-F and MHCD-R were chosen as the template-specific primers since the aim was to amplify all the functional allelic variation present in the population. Forward (5’-CGTATCGCCTCCCTCGCGCCATCAG - MID - TTMYGGCTGTGACCTCCTG-3’) and reverse (5’-CTATGCGCCTTGCCAGCCCGCTCAG - MID - TTGCGCTYCAGCTCTTTC-3’) fusion primers were designed by adding a GS FLX Titanium Primer sequence and a 10bp MID sequence to the 5’ end of the template specific primers (underlined) [46]. The MID’s are the sequence tags that allow individual identification; when both forward and reverse primers are tagged, individual identification can easily be achieved by selecting different combinations of MID’s for each sample. Hence a small number of MID’s were used to uniquely barcode a large number of individuals [28]. We designed ten forward and ten reverse fusion primers using the first ten MID’s from the Standard 454 Set. These MID’s have been engineered by Roche to avoid misassignment of reads and they are tolerant to several errors [46]. The fusion primers were purified using HPLC to minimize the risk of mispriming events [47]. Recently, Lenz and Becker [48] compared the effects of different PCR conditions on *Mhc* artefact formation and showed that simple adjustments like decreasing PCR cycle number or increasing elongation time within each cycle reduced the number of artefacts. As proposed in [48], we tested the minimum number of cycles needed in this study by agarose gel electrophoresis, and found that bands were visible after 28 cycles. Therefore we adjusted the PCR cycle number to 28 and elongation time to 1 minute, to minimize the formation of PCR artefacts. Amplifications were run in a final volume of 25 μl, including 1x FastStart Buffer #2, 0.4 μM of forward and reverse titanium fusion primers, 0.5 units of FastStart Taq DNA Polymerase (Roche), 0.2 mM of each dNTP and 2 μl of extracted DNA (initial concentration of ~5-20 ng/μl) [49]. PCR conditions included an initial denaturation at 94°C for 3 min, followed by 28 cycles of denaturation at 94°C for 15 sec, annealing at 59°C for 45 sec and extension at 72°C for 1 min with a final extension step at 72°C for 8 min.

PCR products were purified using the MinElute 96 UF PCR Purification Kit (Qiagen) and viewed on agarose gels to estimate the concentration of the amplicons. Kloch et al. [30] found this method to be cheaper, less-time consuming and as satisfactory as Nanodrop measurements. 96 individuals with different tag combinations were pooled together in approximate equimolar quantities, and a single pool was prepared for each region of the Pico Titer Plate. The pools were then sent for bidirectional 454 pyrosequencing using GS FLX Titanium chemistry at Genomic Services, Wellcome Trust Centre for Human Genetics, University of Oxford.

### Bioinformatics and data processing

The experiment was run twice for bidirectional sequencing and the results were merged prior to analysis. The output was initially analyzed using jMHC, a software package specifically designed for *Mhc* amplicon analysis [50]. jMHC allows users to specify the template-specific primers, sequence tags, and the individuals represented by each tag. We used the software to extract the reads bearing complete primers and sequence tags; and to generate a table of all variants, and the number of variants represented in each individual. Therefore jMHC sorted out the reads and assigned them to the individuals, and then removed the sequences lacking complete primers or tags, and sequences that had ambiguous base pairs (Ns).

There are three potential sources of error during PCR and pyrosequencing: (i) PCR-generated mutations; (ii) PCR-generated chimeras and (iii) 454 sequencing errors. Most of the 454 sequencing errors constitute over- or under-calls and are low in frequency, hence they are easy to identify [24]. In contrast PCR-generated errors are harder to distinguish as they might occur early in amplification, can have higher frequencies, and the same chimeras may be produced repeatedly (discussed in [24,25,28]). Considering the high number of artefacts generated during these processes, an efficient quality control was crucial to reliably differentiate real alleles from artefacts. We applied a stepwise variant validation procedure to detect true alleles (Table 2); the method is broadly based on the procedures defined by Zagalska-Neubauer et al. [23] and Galan et al. [28].

**Table 2:** Rationale for each step of the variant validation procedure

** Variant validation procedure**	**Rationale**
1 Remove variants that don’t match the expected allele size (212, 215, 221 bp)	Variants that have deletions/substitutions shifting the reading frame probably result from sequencing errors (Assumption 1)
2 Remove variants that have less than four copies in the whole dataset	Variants represented once in an individual probably result from sequencing errors (Assumption 4) and variants represented only in one individual probably result from PCR errors (Assumption 5)
3 Remove individuals with less than 200 reads	A low number of reads per individual might lead to incomplete genotyping, thus the results would be unreliable (Assumption 6). The minimum number of reads required per individual is estimated using the probability distribution plotted by Galan et al. [28]
4 Remove variants that have MPAF lower than 0.01	Variants represented rarely in the whole dataset probably result from sequencing errors (Assumption 2)
Remove variants that have MPAF between 0.01 - 0.025 if they can be explained as a chimera or a single basepair mutation	Variants represented rarely in the whole dataset but more frequently in per individual bases probably result from PCR errors if the parental sequences are also present (Assumption 3)
5 Remove variants that have a single copy per individual	Variants represented once in an individual probably result from sequencing errors (Assumption 4)
Remove variants that have less than five copies per individual if they can be explained as a chimera or a single basepair mutation	Variants represented two, three or four times within an individual probably result from PCR errors if the parental sequences are present (Assumption 3). The threshold for PCR errors is estimated from the distribution of artefacts in the previous step

Variant validation was based on six assumptions: (1) Sequences that have deletions or substitutions shifting the reading frame should be regarded as artefacts; (2) 454 sequencing artefacts should have low copy numbers in the whole dataset and on a per individual basis; (3) PCR-generated mutations and chimeras could have high copy numbers on per individual bases but should co-occur with the allele or allele pair from which they originated [23,48], and should have low copy numbers in the whole dataset; (4) Verified sequences should be represented at least twice in an individual; (5) Verified sequences should be found in at least two individuals; and (6) Reliability of individual genotypes should be ensured by requiring a minimum number of sequences per individual.

Recently, Galan et al. [28] developed a probabilistic model to validate *Mhc* variants and to determine the confidence level of genotyping for each individual. The confidence level (*f*) depended on the values of *r*, *n* and *m*; *r* being the minimum copy number of each true variant, *n* the total number of sequences and, *m* the maximal number of variants for the gene; while the program permitted the presence of 1–4 loci. Although the number of *Mhc* class I loci in great tit was unknown and most likely exceeded 4 loci, we used the program ‘Negative Multinomial’ to estimate the minimum number of sequences required per individual for reliable genotyping and calculated the number of sequences that are necessary for amplifying all the variants at least twice (*r* = 2), giving a confidence level (*f*) of 0.95 [28]. We found that the minimum number of sequences (*n*) required per individual was 9, 23, 39 and 55 for 1, 2, 3 and 4 loci (*m*) respectively. The increase in sequence number was linear with respect to locus number; therefore we estimated that 151–199 sequences would be necessary to genotype 10–13 loci. Previous passerine studies demonstrated the presence of 5–10 *Mhc* class I loci [17,20,43]; hence we chose 200 reads for minimum coverage and two copies per individual for keeping a variant in our study. In Galan et al. [28] the minimum copy number of each true variant was set as three (*r* = 3) and the confidence level was set as 99.9% (*f* = 0.999), hence the minimal number of sequences required for reliable genotyping was much higher, compared to our estimates. Although it would have been ideal to apply the same criteria considering its robustness, in the present study we modified the values because the average number of reads per individual was lower than expected. Therefore we lowered the minimum copy number of each true variant to two (*r* = 2) and the confidence level to 95% (*f* = 0.95) to balance the tradeoff between read quality and size of the dataset. Keeping variants that occur twice in an individual is relatively conservative, but it ensures the retention of real variants and allows the elimination of artefacts in the following steps. It can also be argued that assumptions 4 and 5 are not strictly true and might lead to the removal of rare variants. However we believe that it is better to be conservative and risk missing a small fraction of real alleles, rather than risking the inclusion of artefacts. Therefore our approach was in line with the standard two-PCR criterion – to be verified a sequence should be retrieved at least twice from independent PCR reactions - that *Mhc* studies traditionally use.

**Figure 2:**
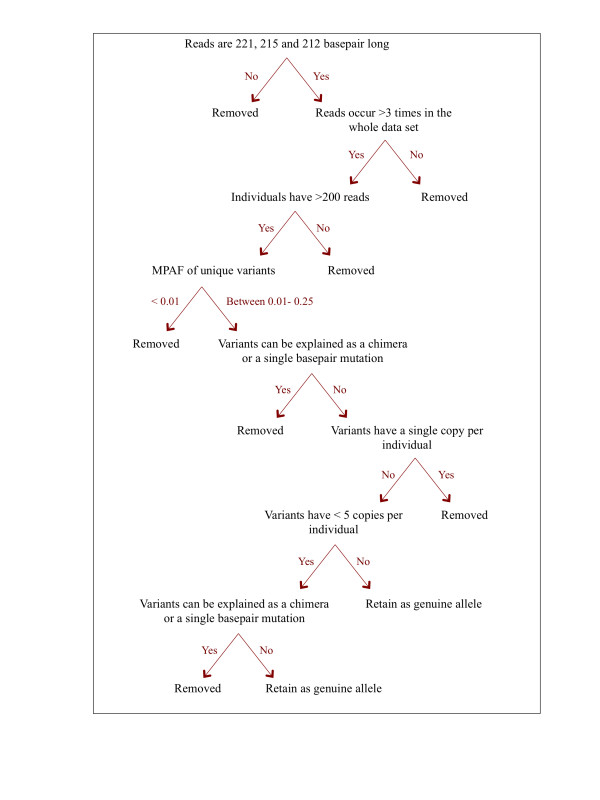
Flow chart of the stepwise variant validation procedure.

The sequence validation procedure had five steps (see Table 2 and Figure 2):

(1)  Reads that did not match the expected allele sizes were eliminated (Assumption 1)

(2)  Reads that occurred only one, two or three times in the whole dataset were removed (Assumption 4 and 5)

(3)  Individuals with fewer than 200 reads were removed (Assumption 6)

(4)  Artefacts were identified at the level of the whole dataset by applying the following criteria:

 Maximum per amplicon frequency (MPAF) was determined for each unique variant [23]. All the variants that had MPAF equal to 0.01 (for these variants the highest within individual frequency was 1%) were examined to detect whether they were real alleles or artefacts. The decision was based on the three individuals that had the highest MPAF for the variant in question. If the variant could be explained as a chimera of more common alleles, or if it differed by a single basepair from more common alleles in all three individuals, it was eliminated as an artefact. Otherwise it was retained as a real allele (Assumption 2 and 3). A total of 82 out of 88 variants (93.2%) with 0.01 MPAF were detected as artefacts and deleted. Then we randomly selected 50 variants with MPAF smaller than 0.01 to check their status; all were identified as artefacts. Therefore we deleted all the variants below 0.01 MPAF. The variants above 0.01 MPAF were examined and the following pattern was detected: 83.4% of the variants (226 out of 271) that had MPAF between 0.011-0.015 were detected as artefacts; 34.5% of the variants (30 out of 87) that had MPAF between 0.016-0.02 were detected as artefacts; but only 2.8% of the variants (2 out of 72) that had MPAF between 0.021-0.025 MPAF were detected as artefacts. The rest of the variants were not checked to prevent false negatives – assigning real alleles as artefacts due to sequence similarities. However we randomly chose 50 variants with MPAF larger than 0.025 and all were identified as real alleles. In total we found and removed 340 artefacts on the scale of the whole dataset. Of these, 96.5% (328 artefacts) had two, three or four copies within the individual, whereas 3.5% (12 artefacts) had five or six copies. Therefore variants that have two, three and four copies within an individual represented the “grey area” as these sequences were likely to be artefacts.

(5)  Artefacts were distinguished at the individual level by applying the following criteria:

 All single copy variants within an individual were deleted (Assumption 4). All variants represented by two, three and four copies were examined to detect whether they could be explained as a chimera of more common variants or whether they differed by a single basepair from more common variants, and were deleted if the answer was yes (Assumption 3). Variants with more than four copies were retained as true alleles.

The fourth and fifth steps of the sequence validation procedure were based on the method developed by Zagalska-Neubauer et al. [23]. These steps involved visual analysis of variants that have low read number in the whole dataset and on a per individual basis, and were critical for distinguishing true alleles from artefacts. Although we increased the risk of introducing artefacts in Step 3 by choosing 200 reads for minimum coverage and two copies per individual for keeping a variant, the detailed inspection of variants in step 4 and 5 enabled us to detect and eliminate errors, hence we believe that the reliability of the genotyping method improved substantially following these two steps.

### Phylogeny construction

A phylogenetic tree of great tit *Mhc* class I exon 3 variants were constructed using Neighbour-Joining method and Tamura-Nei model. The tree was rooted with a chicken (*Gallus gallus*) *Mhc* class I sequence [GenBank: AY234770] and the reliability of the branches was tested with 1000 bootstrap replicates. The phylogenetic tree was constructed using MEGA 5 [51]. The nucleotide diversity between sequences was analyzed using DnaSP ver. 5.10.00 [52].

### Tests of historical selection

The strength of historical selection acting on great tit *Mhc* was tested using a likelihood ratio modelling approach. Two models of codon evolution were compared: the nearly neutral model (M7) where dN/dS < 1; and the positive selection model (M8) where the presence of dN/dS > 1 sites are allowed. If M8 fits the data better than M7, positively selected sites (PSS) were identified through the Bayes empirical Bayes (BEB) procedure. The analysis was computed using CodeML, implemented in the software PAML [53]. The frequencies of dN and dS were then estimated using a second approach: Nei-Gojobari method and Jukes-Cantor correction. The two approaches incorporate different evolutionary models; hence codon based Z-test of selection was conducted on (a) PSS, (b) non-PSS, (c) ABS, (d) non-ABS and (e) all sites, for comparative purposes [31]. The aminoacids corresponding to ABS were detected by superimposing the chicken major *Mhc* class I sequences and assuming concordance at the ABS [54]. The Z-test of selection estimates dN-dS and computes a 1-tailed test to determine if dN > dS. For sites predicted to be in contact with antigens, a significant dN > dS (positive selection) would be expected. Analyses were computed using MEGA 5 [51].

### Definition of supertypes

*Mhc* alleles with similar antigen binding motifs were clustered into supertypes as proposed by Doytchinova and Flower [55]. Initially the aminoacid sequences of all PSS were aligned and the rest of the sites were removed [31,32]. Then each PSS aminoacid was characterized by five physicochemical descriptor variables: *z1* (hydrophobicity), *z2* (steric bulk), *z3* (polarity), *z4* and *z5* (electronic effects) and translated into a matrix [56]. This matrix was subjected to K-means clustering algorithm and model selection for identifying genetic clusters, and discriminant analysis of principal components (DAPC) for describing the clusters using ‘adegenet’ package in R [57,58]. Initially, the function ‘find.clusters’ was used; K-means was run sequentially with increasing number of groups, and different clustering solutions were compared via Bayesian Information Criterion (BIC) values (reviewed in [58]). The number of clusters was then chosen based on the graph of BIC values for increasing number of clusters. The optimal number of supertypes was defined as the minimal number of clusters after which the BIC decreases by a negligible amount and was indicated by an elbow in the curve of BIC values as a function of cluster number. Once the number of clusters was chosen, we applied DAPC to visualize the relationship between the supertypes. The function ‘dapc’ was used to perform a discriminant analysis on the retained principal components (PCs); and a DAPC scatterplot was displayed to illustrate the differences between the clusters using the first two PCs [58].

## Results

### Characterization of great tit *Mhc* class I exon 3

Amplification of genomic DNA using nine different primer pair combinations yielded three distinct PCR products that differed at the length of exon 3 (282 bp, 276 bp or 273 bp). Alleles of similar sizes generally grouped together within the phylogenetic tree, however they were not organized in well supported clusters so it was not possible to identify exact gene families. In total 66 highly divergent alleles were retrieved from 12 great tits (nucleotide diversity = 0.114 ± 0.004). Only three of these alleles were clearly non-functional: their amino acid translation bore two stop codons, and possibly belonged to a pseudogene, as they formed a monophyletic cluster ([see Additional file 1: Figure S1]). Some individuals had up to nine alleles so we estimated that (on the basis of these data alone) the great tit had at least five *Mhc* class I loci, of which one is a pseudogene.

In order to understand which allelic clusters were functional and expressed, we amplified transcribed *Mhc* alleles from mRNA extracted from blood. Contrary to our expectations, the cDNA sequences were not confined to any allelic cluster and were distributed throughout the phylogeny ([see Additional file 1: Figure S1]). Moreover we found that even the pseudogene alleles were being transcribed, as they were present in the cDNA library. Since it was not possible to determine an allelic subgroup of interest for *Mhc* genotyping, we designed the degenerate primers MHCD-F and MHCD-R that would potentially amplify all the functional variation present in great tits. The primers were specifically designed to prevent a good match with the pseudogene alleles, so that the coverage of non-functional sequences could be limited while amplifying the rest of the alleles. The PCR products were 221, 215 and 212 basepair long and covered the major part of *Mhc* class I exon 3 (78%). The primers succeeded in amplifying alleles from each well-supported allelic cluster, suggesting that full scale sequencing of *Mhc* class I alleles could be attained with their use.

## 454 genotyping

Genomic DNA from 1492 individuals, 40 of them amplified twice, and cDNA libraries from four individuals were amplified using the ten forward and ten reverse Titanium Fusion Primers we designed. Bidirectional sequencing was employed using the 16 regions of a Pico Titer Plate gasket. Overall, the experiments generated 638,501 reads. jMHC removed the imperfect sequences reducing the read number to 439,284 (68.8% of the previous step) with a mean of 286 reads per individual and a median of 252. A high level of variation was observed in the number of reads per individual (standard deviation of read number = 173; range = 4–1219). While more than one quarter of the variation in read number was introduced by the 454 region on which a sample was run (due to optimization problems experienced by the sequencing facility), some variation was also introduced by the concentration differences between purified PCR products.

First we eliminated reads that were not 221, 215 or 212 basepair long, since the characterization phase allowed us to define the expected allele sizes, leaving 366,231 reads (83.4% of the previous step) and 83,495 unique variants. Reads that occurred only once, twice or three times in the whole database were then eliminated, reducing the dataset to 332,135 reads (90.7% of the previous step) and 4,674 unique variants. At this stage we removed all the individuals that had fewer than 200 reads, as their genotypes were considered incomplete and thus unreliable. Lastly, artefacts were differentiated at the level of the whole dataset and the individual, resulting in retention of only 862 variants as true alleles. Overall, 871 samples passed our criteria: 857 individuals, 12 duplicates and two cDNA amplicons; the final genotypes were based on 214,357 reads (64.5% of the previous step).

**Figure 3:**
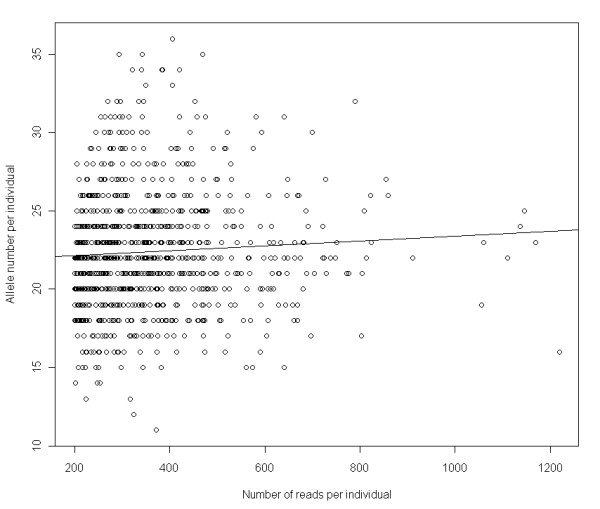
Variation in allele number per individual with increasing read number.

Among the 40 samples genotyped twice only 12 duplicates passed our variant validation criteria, and the reliability of the experiment was calculated based on these samples. We removed the pseudogene alleles that bore a stop codon prior to calculation, because the primers were designed to avoid amplification of such alleles and they were functionally irrelevant. For each duplicate we calculated the agreement between the genotypes after the 3rd, 4th and 5th step of the variant validation procedure, to verify the efficiency of the method (see Table 3). We also calculated the agreement between the genotypes following supertype classification (see Table 3, last column), since supertypes will be considered as the unit of selection in subsequent analyses investigating the effects of *Mhc* variation on parasite burdens, survival, reproductive success and mate choice in this system. On average the repeatability of the duplicates increased from 0.34 to 0.94 between step 3 and step 5; 22 alleles were common in the duplicate, while 1.4 alleles occurred only in one duplicate. Of the 12 individuals six had identical genotypes following variant validation. A repeatability score was calculated for each duplicate and the scores were averaged to estimate the repeatability of the experiment. Hence the repeatability of the genotyping method was calculated as 94%. The lack of significant correlation between read number and allele number per individual, above the threshold of 200 reads (Figure 3) further supported the reliability of the experiment (R^2^ = 0.0038, p = 0.07). The agreement between genotypes increased from 0.94 to 0.96 after supertype classification; on average 10 supertypes were common in the duplicate, whereas 0.4 supertypes occurred in one duplicate. Of the 12 individuals eight had identical genotypes following supertyping and we found no correlation between the read number and supertype number per individual (R^2^ = 0.0006, p = 0.45) ([see Additional file 1: Figure S2]).

**Table 3:** Repeatability measures for each duplicate pair after 3rd, 4th and 5th step of the variant validation procedure and following supertype classification

	**Read no**	**Verified alleles / ST**				**Unverified alleles / ST**				**Repeatability**			
		**S3**	**S4**	**S5**	**ST**	**S3**	**S4**	**S5**	**ST**	**S3**	**S4**	**S5**	**ST**
**duplicate-1**	528 - 668	31	28	28	9	120	10	0	0	0.21	0.74	1	1
**duplicate-2**	387 - 418	29	29	28	11	71	14	0	0	0.29	0.67	1	1
**duplicate-3**	322 - 501	22	22	22	13	54	6	0	0	0.29	0.79	1	1
**duplicate-4**	471 - 507	26	25	22	9	71	7	0	0	0.27	0.78	1	1
**duplicate-5**	267 - 484	21	21	21	8	40	6	0	0	0.34	0.78	1	1
**duplicate-6**	276 - 456	21	20	20	10	52	4	0	0	0.29	0.83	1	1
**duplicate-7**	288 - 319	23	23	22	10	32	4	1	0	0.42	0.85	0.96	1
**duplicate-8**	208 - 455	21	21	20	10	50	13	2	0	0.3	0.62	0.91	1
**duplicate-9**	272 - 289	27	26	23	10	34	3	3	1	0.44	0.89	0.88	0.91
**duplicate-10**	240 - 471	21	21	18	10	66	20	3	1	0.24	0.51	0.86	0.91
**duplicate-11**	230 - 236	24	24	21	11	26	6	4	1	0.48	0.8	0.84	0.92
**duplicate-12**	240 - 570	20	20	19	10	50	22	4	2	0.29	0.48	0.83	0.83
***Average***	*287 - 448*	*24*	*23*	*22*	*10*	*56*	*9.6*	*1.4*	*0.4*	*0.32*	*0.73*	*0.94*	*0.96*

ST – supertypes; read no - the number of reads per sample. Verified alleles/supertypes stand for the number of identical alleles/supertypes between the duplicate samples. Unverified alleles/supertypes represent the number of alleles/supertypes that were found in one of the samples and indicate non-identical genotypes. Repeatability was calculated as the ratio of verified alleles/supertypes to the total number of alleles/supertypes for each duplicate. A repeatability value of 1 signifies identical genotypes. For a duplicate each measurement was calculated four times - after the 3rd, 4th and 5th steps of the variant validation procedure, and following supertype classification. The average values for each measure are provided in the bottom row.

### Sequence diversity

**Figure 4:**
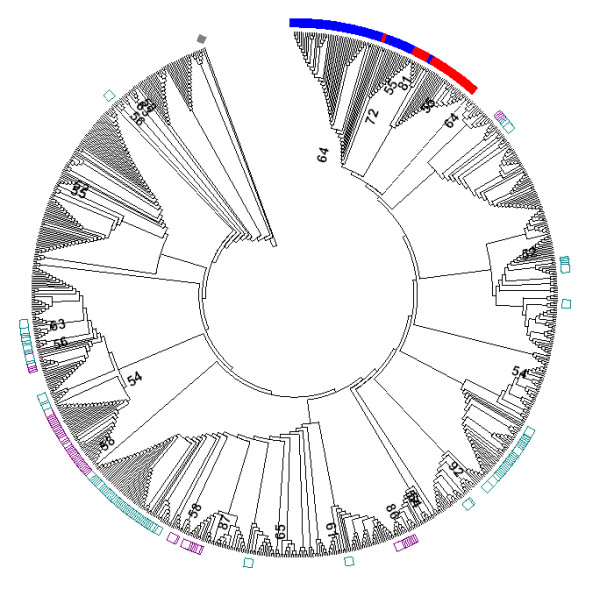
**Phylogenetic tree of great tit *Mhc *class I exon 3 sequences.** The tree was constructed using Neighbour-Joining method and Tamura-Nei model. The sequences [GenBank: JQ034624 - JQ035485] differed at the length of exon 3 being either 221 bp, 215 bp or 212 bp. The tree was rooted with a chicken (*Gallus gallus*) *Mhc* class I sequence [Genbank: AY234770] and the reliability of the branches was tested with 1000 bootstrap replicates. Bootstrap supports for the major clades are indicated with numbers. Pseudogene alleles bearing stop codons are marked in red and putatively non-functional alleles (Group 1) are marked in blue. The rest of the alleles represent Group 2 (except the chicken *Mhc* class I sequence). The chicken *Mhc* allele is marked in grey. The sequences that are 221-basepair long are indicated by purple squares and the sequences that are 215-basepair long are indicated by green squares. The 212-basepair sequences are not marked.

In this study a total of 862 *Mhc* class I alleles [GenBank: JQ034624 - JQ035485] were detected; to our knowledge the highest number characterized in a wild bird species (Figure 4). The sequences were highly divergent with 133 polymorphic nucleotides, representing 60% (133/221) of the sites. Nucleotide diversity and the average number of nucleotide differences were π = 0.106 ± 0.001 and 22.59 ± 0.23 respectively. Of 66 alleles we retrieved in the characterization stage, fifty were present among these sequences (76%). Within the sample of 862 alleles, 39 were non-functional alleles bearing stop codons: 36 of these alleles had two stop codons at the same location, were highly similar to each other and formed a paraphyletic group suggesting the presence of a pseudogene family ([see Additional file 1: Figure S3]). Moreover these sequences formed a monophyletic cluster with 68 alleles that did not bear stop codons, but showed a dissimilar pattern of divergence from the rest of the alleles. These 68 sequences are referred as Group 1, while the remaining 758 sequences are referred as Group 2 (see Figure 4). The members of Group 1 were 215 basepairs long, similar to each other, and structurally similar to the pseudogene alleles variants ([see Additional file 1: Figure S3]). The segregating sites within Group 1 were considerably different than the segregating sites in Group 2, implying that separate evolutionary forces might be shaping this variation and that Group 1 sequences might be representing non-functional variants. Alleles of Group 2 were, on the other hand, highly divergent from each other, had similar segregating sites and varied in their size (221, 215 and 212 basepair long). Three non-functional alleles bearing stop codons (of the 39) were within Group 2, but unlike the members of the pseudogene family these sequences bore only one stop codon and were not clustered together, implying that they evolved independent from each other ([see Additional file 1: Figure S1]). These alleles were probably formed recently as a result of mutation accumulations, and did not belong to a pseudogene.

Two cDNA amplicons passed our variant validation criteria and 42 alleles were retrieved in total. We checked whether the cDNA alleles were confined to particular allelic clusters; however, in line with our previous findings, the sequences were found throughout the phylogenetic tree (including the pseudogene family) and did not fall into any cluster. Hence the sequence variants obtained from cDNA were not useful in differentiating functional groups from the non-functional ones.

### Signatures of historical selection

**Figure 5:**
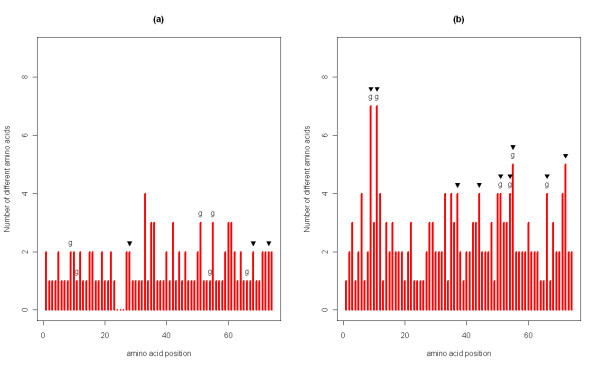
**Aminoacid variation plot for (a) Group 1 and (b) Group 2 alleles.** Chicken antigen binding sites (ABS) are indicated with the letter ‘g’, whereas positively selected sites (PSS) are indicated with black triangles. In the Group 1 plot there are no aminoacids between the positions 24–26, because these alleles were 212-basepair long; hence had a nine-basepair deletion at this location.

Tests of historical selection were performed separately on Group 1 and Group 2 alleles in order to clarify whether they were under different selective pressures. First we used a likelihood ratio modelling approach to compare the two models of codon evolution (nearly neutral versus positive selection) and selected the model that best fitted the data (see Table 4 for the results of the likelihood ratio tests). For Group 1 the model allowing a class of sites to be under positive selection (M8) fitted the data better than the null nearly neutral model (M7). Therefore M7 was rejected in favour of M8, and three sites were found to be under positive selection according to Bayes empirical analysis (P < 0.05 for all three sites). However none of these sites were identical to the chicken ABS, and only one was situated close to an ABS with a distance of two aminoacids (Figure 5a). For Group 2 it was not possible to run the analysis on all of the alleles because the number of sequences exceeded the capacity of CodeML software. Therefore we selected all the sequences that were present in more than 20 individuals as being common alleles, confirmed that they were equally distributed throughout the phylogeny ([see Additional file 1: Figure S4]) and ran the analyses on these 117 sequences. Again M8 fitted the data better and nine sites were detected as PSS (P < 0.05 for all nine sites). Six PSS were identical to the chicken ABS, suggesting full agreement (Figure 5b). The remaining three PSS had high aminoacid variability and might have been sites of peptide or T cell binding. None of the PSS identified in Group 2 were identical with the PSS in Group 1, confirming that these two groups are under different selection pressures, and hence probably differ in their functionality.

**Table 4:** Results of the likelihood ratio tests for different models of codon evolution and estimated parameter values

**Model**	**lnL**	**ΔAIC**	**Parameters**
	**Group 1**		
M0 – one ω	−2975.2	454.8	ω = 0.53
M7 – nearly neutral with β	−2788.9	100.2	
M8 – positive selection with β (ω_0_ ≤ 1, ω_1_ >1)	−2737.8	Best	*p*_0_ = 0.83, *p*_1_ = 0.17, ω_1_ = 2.92
	**Group 2**		
M0 – one ω	−1154.5	68.5	ω = 0.74
M7 – nearly neutral with β	−1124	25.5	
M8 – positive selection with β (ω_0_ ≤ 1, ω_1_ >1)	−1110.2	Best	*p*_0_ = 0.93, *p*_1_ = 0.07, ω_1_ = 5.26

ω - dN/dS; nearly neutral with β - for all sites dN/dS ≤ 1 and the beta distribution approximates ω variation; positive selection - a proportion of sites evolves with dN/dS > 1; lnL - Log-likelihood value; ΔAIC - the difference between the value of the AIC of a given model and the best model; *p*_0_ - proportion of sites with dN/dS ≤ 1, *p*_1_ - proportion of positively selected sites (dN/dS > 1), ω_1_ - estimated value of ω for sites under positive selection.

We also performed codon based Z-test of selection on Group 1 and Group 2 separately (Table 5). Here we checked whether PSS, non-PSS, chicken ABS, chicken non-ABS and all sites were under selection to validate the results of the previous analysis. As expected, none of the sites were found to be under positive selection in Group 1, whereas both PSS and chicken ABS (sites predicted to be involved in antigen recognition) were positively selected in Group 2.

**Table 5:** Results of codon based Z- test of selection for group 1 and group 2

	**n**	**dN**	**dS**	**Z**	**P**
	**Group 1**				
**ABS**	18	0.171 ± 0.135	0.016 ± 0.03	1.244	0.108
**Non-ABS**	194	0.067 ± 0.014	0.112 ± 0.034	−1.218	1
**PSS**	9	0.152 ± 0.101	0.038 ± 0.039	1.493	0.069
**Non-PSS**	203	0.065 ± 0.014	0.115 ± 0.036	−1.254	1
**All**	212	0.075 ± 0.015	0.103 ± 0.032	−0.788	1
	**Group 2**				
					
**ABS**	18	0.578 ± 0.132	0.134 ± 0.068	3.694	**<0.001**
**Non-ABS**	203	0.054 ± 0.011	0.128 ± 0.032	−2.106	1
**PSS**	27	0.492 ± 0.092	0.069 ± 0.051	5.801	**<0.001**
**Non-PSS**	194	0.043 ± 0.009	0.136 ± 0.034	−2.686	1
**All**	221	0.085 ± 0.017	0.126 ± 0.029	−1.158	1

The rates of non-synonymous mutations (dN) and synonymous mutations (dS) were computed using Nei-Gojobari method and Jukes-Cantor correction. The standard errors were obtained through 1000 bootstraps replicates. Z test of selection estimated dN-dS (indicated as Z) and computed a1-tailed test to determine whether dN > dS (indicated as P). n is the number of nucleotides representing antigen binding sites (ABS), non antigen binding sites (Non-ABS), positively selected sites (PSS), non positively selected sites (Non-PSS) and all sites in Group 1 and Group 2. ABS were detected by superimposing the chicken *Mhc* class I sequences and assuming concordance. Significant p-values are bold.

### Supertypes

**Figure 6:**
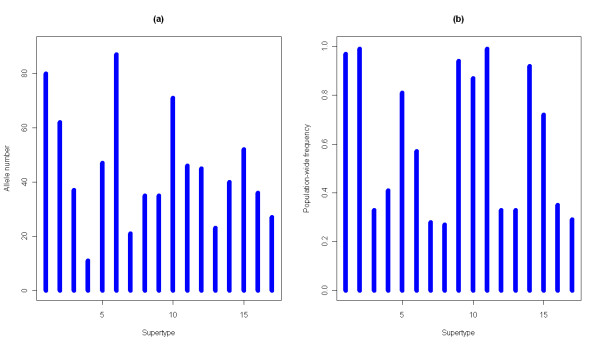
**Plot of (a) *Mhc *allele number per supertype and (b) frequency distribution of supertypes.**

**Figure 7:**
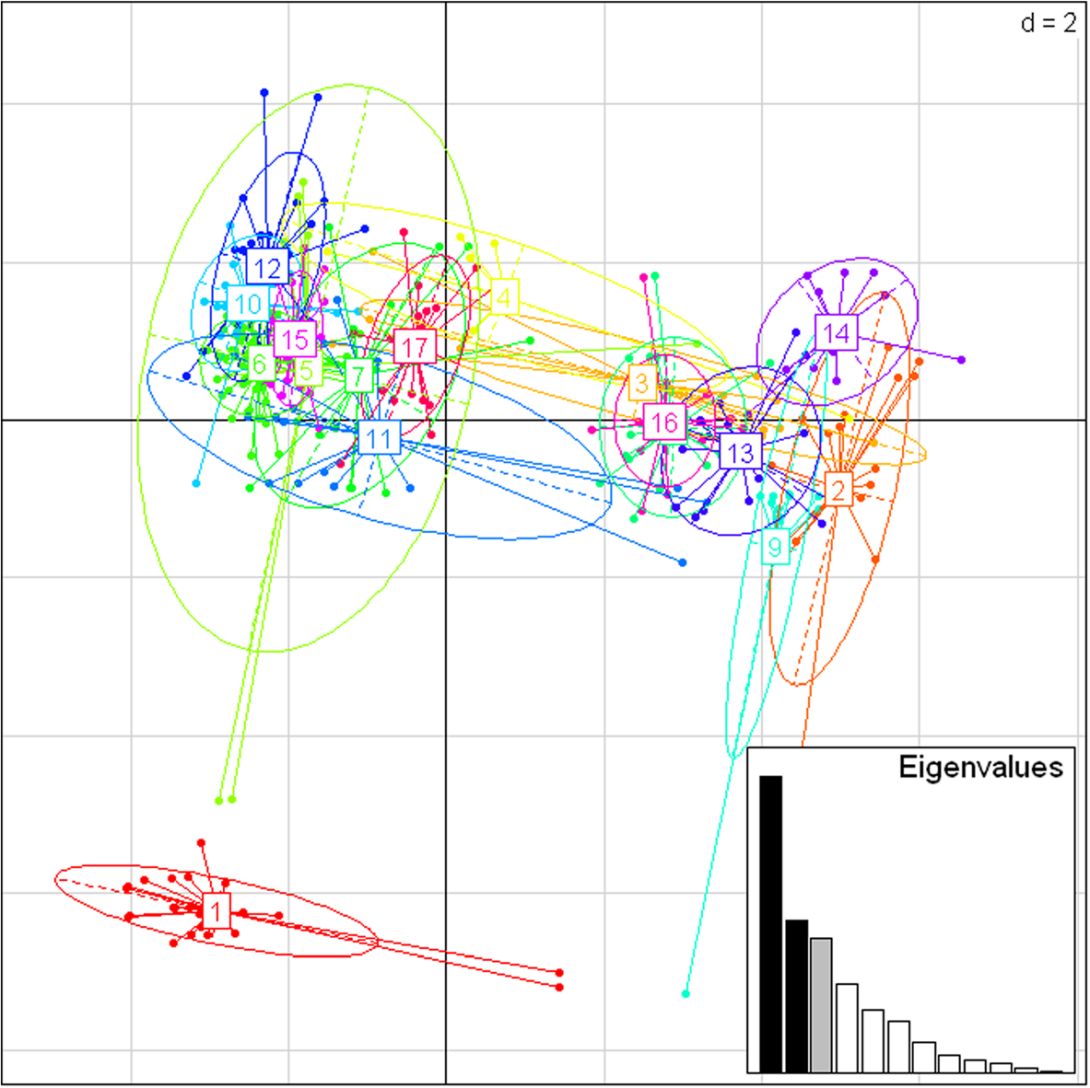
**DAPC scatterplot of the 17 *Mhc *supertypes.** 12 PCs and three discriminant functions (dimensions) were retained during analyses, to describe the relationship between the clusters. The scatterplot show only the first two PCs (d = 2) of the DAPC of *Mhc* supertypes. The bottom right graph illustrates the variation explained by the 12 PCs. Each allele is represented as a dot and the supertypes as ellipses.

Group 2 sequences were clustered in functional supertypes, using the nine sites detected as PSS. We excluded the alleles bearing stop codons and the Group 1 sequences because they were considered non-functional. We created a matrix for the 775 Group 2 alleles based on their PSS physicochemical properties; and subjected the matrix to K-means clustering algorithm and model selection. The optimal number of clusters was indicated by a change in the slope of BIC decrease (the elbow in the curve) and was identified as 17 ([see Additional file 1: Figure S5]). Allele number per supertype ranged from 11 to 87 alleles with a mean of 44 (Figure 6a), and the population-wide frequency of supertypes ranged from 0.27 to 0.99 (Figure 6b). Once the number of clusters was chosen, we applied DAPC to visualize the relationship between the 17 supertypes. For this analysis 12 principal components (PCs) were kept to retain 93% of the variation. The DAPC scatterplot summarizes the differences between the clusters using the first two PCs (Figure 7).

### Estimating the number of *Mhc* class I loci

The maximum number of alleles per individual was found to be 37 with a mean of 23.8 ± 3.9, suggesting that there are at least 19 *Mhc* class I loci in the great tit (Table 6). However some of these alleles belonged to a pseudogene, whereas others were considered non-functional (Group 1 alleles). We found a maximum of 32 functional alleles per individual with a mean of 19.5 ± 3.7. Therefore we estimate that the great tit has at least 16 functional loci. When functionally similar alleles were clustered in discrete supertypes, each individual had between 6–16 supertypes with a mean of 10.4 ± 1.6; the number of supertypes was approximately normally distributed in the population. Based on the data, the number of pseudo and putatively non-functional genes were also estimated. These numbers are probably an underestimate as the primers were designed to prevent a good fit with the alleles bearing a stop codon, and the putatively non-functional alleles were similar to the pseudogene alleles. Nevertheless, we found a maximum of six pseudogene alleles and six putatively non-functional alleles per individual, pointing to the presence of at least three loci in each group.

**Table 6:** **Summary of *Mhc *genotyping results and estimation of minimum loci number**

	**Range**	**Mean**	**s.d.**	**Number of loci**
Number of alleles per individual	12 - 37	23.76	3.98	19
Number of functional alleles per individual	9 - 32	19.48	3.74	16
Number of supertypes per individual	6 - 16	10.36	1.62	-

s.d.- standard deviation. Minimum loci number was estimated using the maximum allele number for each class.

## Discussion

In this study, characterization of great tit *Mhc* class I exon 3 was adopted and large-scale, high-resolution genotyping was carried out by the use of 454 pyrosequencing method. A total of 862 alleles with varying sizes and high nucleotide diversity were detected in 857 great tits, demonstrating that this species has a polymorphic, multilocus *Mhc* region; tests of historical selection implied this polymorphism was mainly maintained by balancing selection. Full analysis of sequences through a stepwise variant validation procedure allowed reliable typing of 871 samples and duplicates confirmed the repeatability of the genotyping method. Lastly, the effective *Mhc* repertoire of each great tit was described by clustering alleles with similar antigen binding affinities into 17 supertypes.

Our characterization effort proved to be effective as it enabled us to define the allelic groups of interest and the sequences to prevent (pseudogenes) during 454 pyrosequencing. High diversity among the potentially functional alleles led to the design of a degenerate primer pair, however attention was paid to minimize amplification of alleles with stop codons. Consequently, functional sequences constituted the major fraction of the reads (87%) that were retained following variant validation procedure, whereas the number of reads belonging to putatively non-functional genes were much lower. This highlights the importance of carrying out a thorough background work prior multilocus typing, not only to attain an initial understanding of gene and allelic diversity, but also to design the ideal primers that target the expressed alleles only. For instance, Zagalska-Neubauer et al. [23] used 454 pyrosequencing to type the *Mhc* class II region of the collared flycatcher and the experiment successfully generated a mean of 541 reads per individual. However the coverage was insufficient for reliable genotyping since the primers extensively amplified the putative *Mhc* pseudogenes, greatly decreasing the coverage of expressed alleles.

We retrieved 76% of the alleles identified during the characterization stage. These were randomly distributed in the phylogeny and represented each allelic group, suggesting that we successfully amplified the allelic groups of interest. Few of the alleles that were not retrieved belonged to Group 1 or the pseudogene group as expected. The rest of the alleles identified at the initial characterization stage were most likely PCR-generated errors that were not differentiated at the characterization stage as they were either 1 basepair different from more common alleles or they could be explained as a chimera of more common alleles. Lenz and Becker [48] showed that PCR generated artefacts could constitute up to 25 percent of the alleles when no approach is taken to reduce artefact formation. Although we adjusted the PCR conditions during amplification for 454 genotyping, we used the standard conditions during characterization. Hence it would not be surprising to have generated a relatively high frequency of artefacts during the cloning –sequencing processes.

We used the 16 regions of the Pico Titer Plate and pooled 96 great tit amplicons in each region to maximize the number of individuals sequenced in the experiment. However from the total of 1536, only 871 samples (56%) were successfully genotyped following the variant validation procedure. The efficiency of the experiment could have been improved by optimising the sample number for a full plate run [28]. In amplicon sequencing, the depth of coverage is tightly linked to the number of amplicons being pooled. Therefore by pooling a smaller number of amplicons, we could have genotyped more individuals. However, it is rather difficult to determine the optimal number of samples to use in a 454 run, especially in cases where the gene copy number is initially unknown. Alternatively, our method could have been improved by calculating the concentration of each amplicon using a Nanodrop, in order to assure the pooling of equimolar quantities [24]. This way the variation in read number per sample could have been minimized and possibly more individuals would have passed the minimum coverage threshold. Due to the error-prone sequencing technology of NGS, we applied a five-step variant validation procedure to differentiate real alleles from PCR/sequencing artefacts. It can be argued that some rare alleles might have been missed in few individuals (especially in the ones that had read numbers slightly exceeding 200) while these steps were being applied. However we believe this is unlikely to be a very large effect given that no significant correlation between read number and allele number per individual was found. Moreover the consistency of the genotyping method and the variant validation criteria was confirmed by the high repeatability (0.94) of duplicates, and by the substantial increase in the repeatability measures between the 3rd and 5th step of variant validation procedure.

Still, of the 12 duplicates only six had identical genotypes following variant validation. Therefore it is plausible to suggest that the quality of the genotyping method could have been improved by modifying the variant validation procedure. As mentioned in the methods section, we tried to maintain a balance between the quality and size of the dataset whilst setting up the genotyping criteria; hence a better experimental design would have maximized both measures. The inconsistency among samples in six of the duplicates was mostly due to removal of real alleles, because the variants in question had low frequency on per individual basis and were highly similar to more common variants. Although the removal of rare alleles poses a problem for accurate genotyping, the application of supertype classification alleviated the issue to some extent, since highly similar alleles were grouped into the same supertype and the contribution of single alleles became irrelevant following supertyping. Hence, of the 12 duplicates eight had identical genotypes after supertyping, no correlation was found between the read number and supertype number per individual and the agreement between duplicates was calculated as 0.96. To date few *Mhc* studies carried out an empirical assessment of genotyping error via running duplicates in non-model vertebrates [24], therefore it is hard to assess how good the agreement is, but we believe that in the context of ecological studies, this level of repeatability is sufficient.

Four great tit cDNA libraries were constructed from mRNA sequences extracted from blood, and the libraries were used to identify transcribed allelic clusters. However a few cDNA sequences were retrieved from each allelic cluster, so it was not possible to isolate any group as more functionally important. Surprisingly, pseudogene alleles that bore stop codons were also found in the cDNA libraries. This emphasized the fact that classical sequencing methods are limited and even problematic if used for expression analysis, as they only inform on the presence or absence of transcripts. Such data are insufficient, because even non-functional genes could be transcribed in low levels unless a mutation at the promotor region halts the transcription completely. Therefore an approach based on expression level quantification (for instance using real-time PCR) should be adopted in order to differentiate highly expressed, hence functionally important alleles [26]. Assessment of gene expression through cDNA coverage has started to find wider application in NGS methods [59].

Alternatively, the presence of non-functional cDNA sequences can also be explained by genomic DNA contamination during RNA extraction. However we believe this is unlikely, because the method used for RNA purification (RNeasy Protect Animal Blood) included a DNase digestion step. Although mRNA data did not inform us on allelic expression, the phylogenetic tree of the class I alleles and the historical selection tests significantly improved our understanding of functional alleles. The Group 1 sequences formed a monophyletic cluster with pseudogene alleles and codon based Z-test of selection suggested none of the putative antigen binding sites were positively selected. Still we detected a weak and non-significant signal for the positive selection of Group 1 ABS. Such weak signals are expected, as it has been shown to take around 19–74 million generations for a positive dN-dS signal to disappear even in the absence of selection [60]. Using a likelihood ratio modelling approach three sites were found to be under positive selection in Group 1, yet these were not within highly variable sites. In contrast all the putative antigen-binding amino acids were under positive selection in Group 2, in addition to three highly variable sites. Moreover Z –test of selection suggested both ABS and PSS were under great selective pressure. These results implied that Group 2 sequences were actively involved in antigen recognition and have been under selection from a variety of parasites; whereas Group 1 sequences were non-functional, hence the observed variation possibly accumulated as a result of relaxed selection.

In total 755 alleles were considered functional and individual great tits possessed 9 to 32 putative expressed alleles. A discrepancy in the number of alleles per individual is common in *Mhc* studies across taxa, due to variation in gene copy number within species and allele-sharing between loci [24,31,61,62]. We cannot exclude the possibility that some alleles might have been missed at the PCR stage, although we believe this to be relatively unlikely considering that we gathered extensive sequence information during characterization, and amplifications were performed using a degenerate primer pair. Similarly sized alleles did not form monophyletic groups implying that 3 bp insertions and deletions occurred several times in the history of the gene, independent of each other. The important contribution of indels to *Mhc* genomic diversity has already been shown in chicken [63]. Moreover a comparative study between human and chimpanzee *Mhc* proposed indels as the major driving force for genomic divergence [64]. Alternatively, this pattern might also be an effect of recombination and gene conversion, processes shown to be frequent in passerine *Mhc* systems [20,23].

The alleles of Group 2 were clustered into 17 functional supertypes based on the physicochemical properties of their PSS. We adopted a bioinformatic approach that was described by Doytchinova and Flower [55] to determine peptide specificity; however an experimental approach would have been ideal. For instance, a recent study established computational antigen-binding prediction algorithms based on empirical datasets, and showed an evolutionary advantage for allele pairs that are more divergent in recognizing a broader range of potential antigens [65]. Moreover the biological relevance of grouping *Mhc* alleles with similar antigen binding affinities into supertypes is supported by a growing number of human and non-human primate studies [33,35,66,67]. In this study we utilized a newly proposed method for identifying genetic clusters (reviewed in [58]). This method is an advance on previous methods, as it does not require arbitrary clustering decisions [32], but uses K-means clustering algorithm and model selection approach to compute associated summary statistics.

We found evidence for the presence of at least 16 functional loci in the great tit. This constitutes the highest number of expressed *Mhc* class I alleles/loci identified in a passerine species. However we believe it is likely that this complexity is not specific to great tits and that similar patterns can be found in other passerines. Studies on great reed warbler and scarlet rosefinch have already revealed high polymorphism and the existence of more than 5–6 functional *Mhc* class I loci in these species, although these studies had lower sample sizes (248 and 120 respectively) and used conformation-based mutation detection methods that rely on physical separation of alleles, without providing allele sequence information [20,43]. The utilization of indirect typing methods and motif-specific primers can be a rewarding approach in species where functional alleles are well described and confined to an allelic subset. However in species where the *Mhc* structure is complex and the number of co-amplifying alleles is high, like in the great tit, it is impossible to differentiate variants based on their migratory patterns and it is inevitable to underestimate the allelic diversity as a result of peak overlapping [28]. Moreover, because indirect typing methods require cloning-sequencing effort to reveal the nucleotide information, hundreds of clones must be sequenced to obtain a good estimate of allelic composition in such complex systems [25]. Therefore it is feasible to suggest that the employment of 454-pyrosequencing is ideal for passerine species that harbour many *Mhc* loci and high allelic diversity, as it has the potential to reveal the extent of *Mhc* complexity. In a recent study 454 technology was used for *Mhc* genotyping Atlantic cod and it was shown that the species possess more than 100 *Mhc* class I loci, a greatly expanded number compared to other teleost fish [68]. This extraordinary expansion of class I genes was explained by the absence of class II loci; this might represent a compensatory mechanism adapted by the Atlantic cod immune system. This work illustrates the great flexibility in *Mhc* genomic organization among closely related species.

## Conclusions

We found extreme complexity at the *Mhc* class I exon 3 of the great tit following a broad characterization and large-scale, high-resolution genotyping effort. The presence of at least 16 functional loci was shown, together with a pseudogene family and putatively non-functional alleles. There was clear evidence that expressed alleles were under strong balancing selection and these alleles were grouped into 17 functional supertypes based on their antigen binding affinities. This study represents the first step of an in-depth analysis of the genetic basis of disease resistance, aimed at better understanding how parasite-mediated and sexual selection shape and maintain host genetic variation in nature. We believe that this approach will find wide application among evolutionary biologists in the near future and will allow advancements in our understanding of complex genetic regions.

## Authors’ contributions

IS participated in study design, carried out the molecular genetics procedures, analyzed the data and drafted the manuscript. HKM provided tools for bioinformatic analyses and helped to draft the manuscript. EH helped with statistical analyses and drafting the manuscript. BCS participated in study design and coordination, provided the samples and helped to draft the manuscript. All authors read and approved the final manuscript.

## Supplementary Material

Additional file 1 **Fig S1, Fig S2, Fig S3, Fig S4, Fig S5. Figure S1. **Phylogenetic tree of the Mhcclass I sequences we identified during characterization. The tree was constructed using Neighbour-Joining method, Jukes-Cantor model and rooted with a Scarlet Rosefinch (*Carpodacus erythrinus*) *Mhc* class I exon 3 sequence [Genbank: FJ392790]. The interior branch numbers refer to bootstrap values with 2000 replications. Blue squares indicate sequences retrieved from cDNA. Three sequences adjacent to the Scarlet Rosefinch *Mhc* allele are the pseudogene alleles and are indicated with the letter ‘P’. **Figure S2.** Variation in supertype number per individual with increasing read number. **Figure S3.** Aminoacid sequences of a representative set of great tit *Mhc* class I alleles (exon 3). The first set of alleles (Pama-U*II001-II758) are members of Group 2; the second set of alleles (Pama-U*I17-I60) are members of Group 1; and the third set of alleles (Pama-U*P15-P34) are members of the pseudogene. Identity with Pama-U*II001 is indicated with dots, differences are shown by letter substitutions, and gaps are shown by dashes. Stop codons are shown by stars. Chicken antigen binding sites (ABS) are shaded with blue, while Group 2 positively selected sites (PSS) are indicated with ‘+’. Pama-U*II758 is one if the three non-functional alleles within Group 2. **Figure S4.** Phylogenetic tree of functional Group 2 sequences. The tree was constructed using Neighbour-Joining method, Jukes-Cantor model and rooted with a chicken (*Gallus gallus*) *Mhc* class I sequence [Genbank: AY234770]. The reliability of the branches was tested with 1000 bootstrap replicates. Bootstrap supports for major clades are indicated with numbers. The alleles that were present in more than 20 individuals are marked in red. The chicken *Mhc* allele is marked in grey. **Figure S5.** Graph of BIC values for increasing number of clusters. The optimal number of supertypes was identified as 17 (marked with a vertical, black dashed-line) as it indicated the elbow in the curve of BIC values. After 17 clusters the slope of BIC decrease dropped notably. The change in the slope at the 17th cluster is indicated with two, red dashed-lines. Click here for file
