# Supplementary figures and images for: Characterization and 454 pyrosequencing of Major Histocompatibility Complex class I genes in the great tit reveal complexity in a passerine system
Source: BMC Evol Biol. 2012 May 15;12:68. doi: 10.1186/1471-2148-12-68 (PMC3483247; doi:10.1186/1471-2148-12-68)

## Slide 1
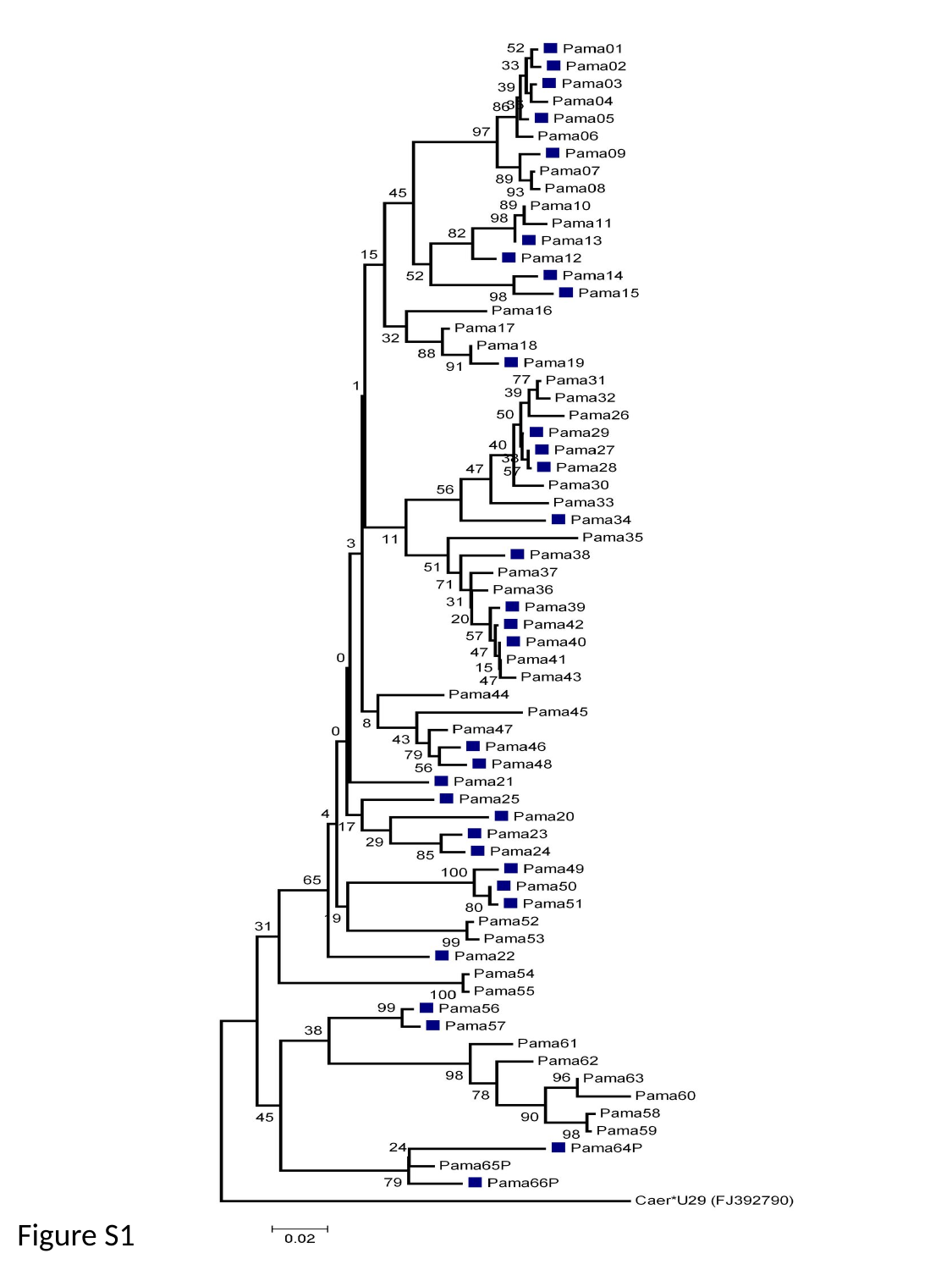

Figure S1

## Slide 2
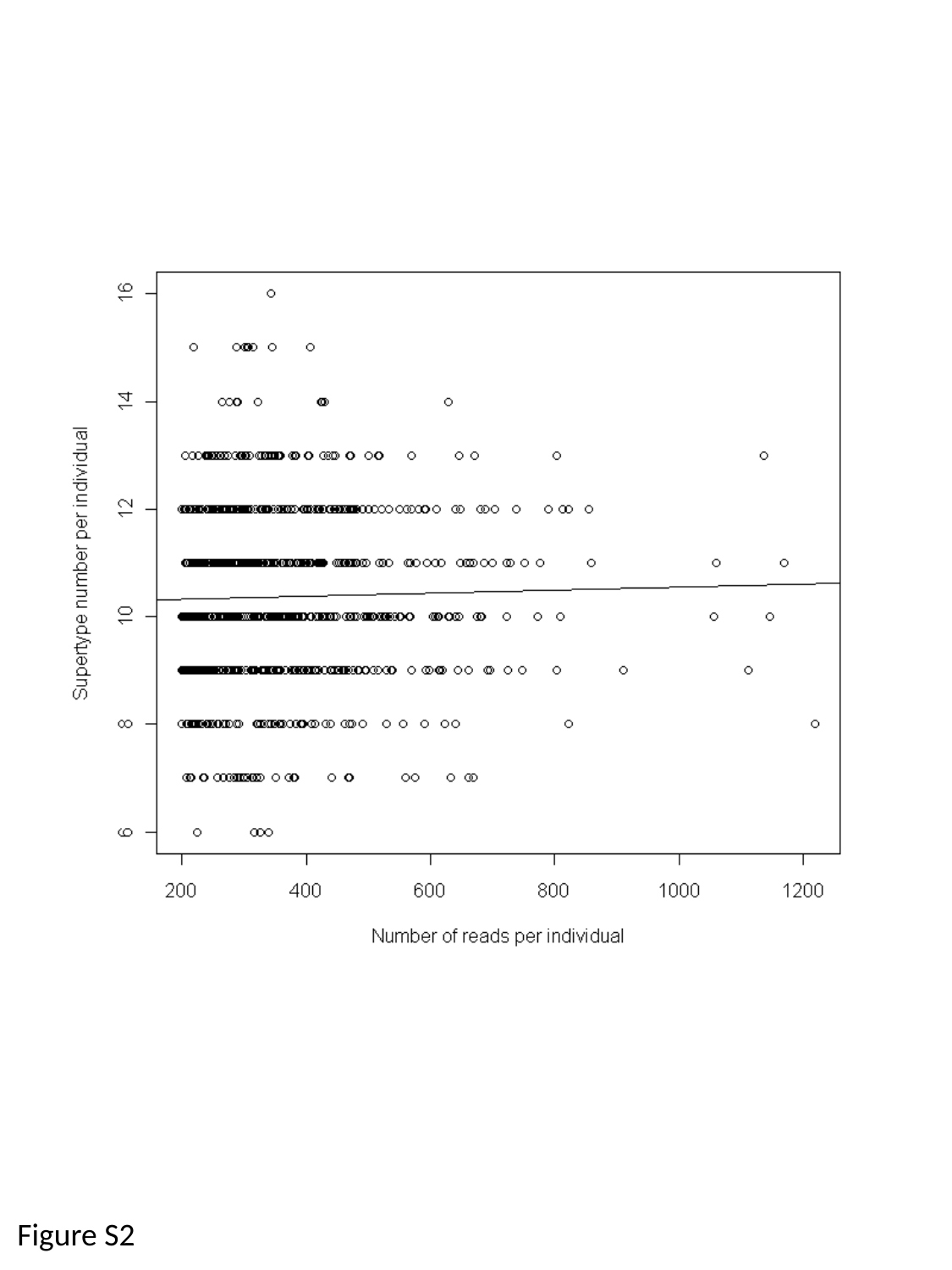

Figure S2

## Slide 3
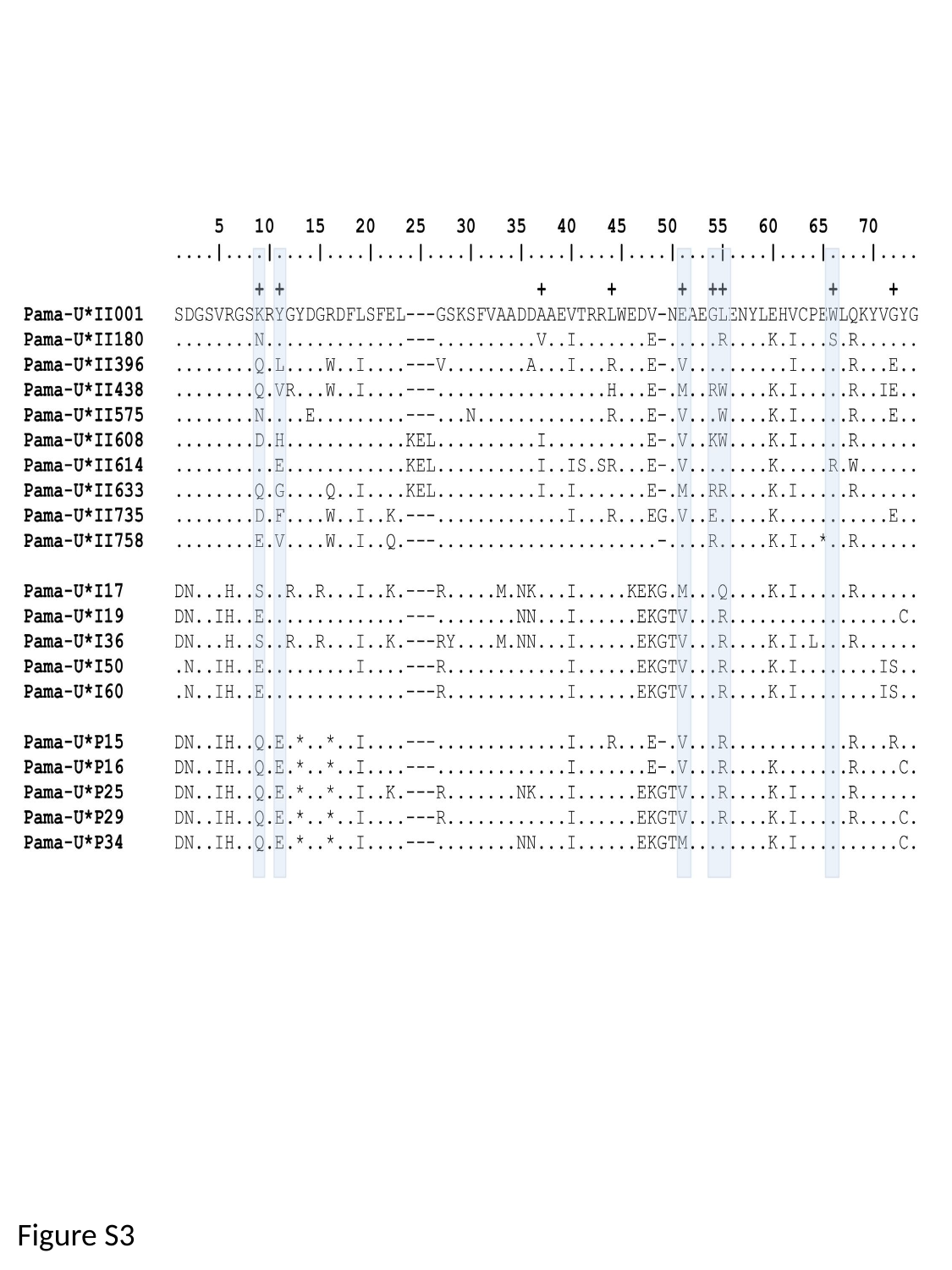

Figure S3

## Slide 4
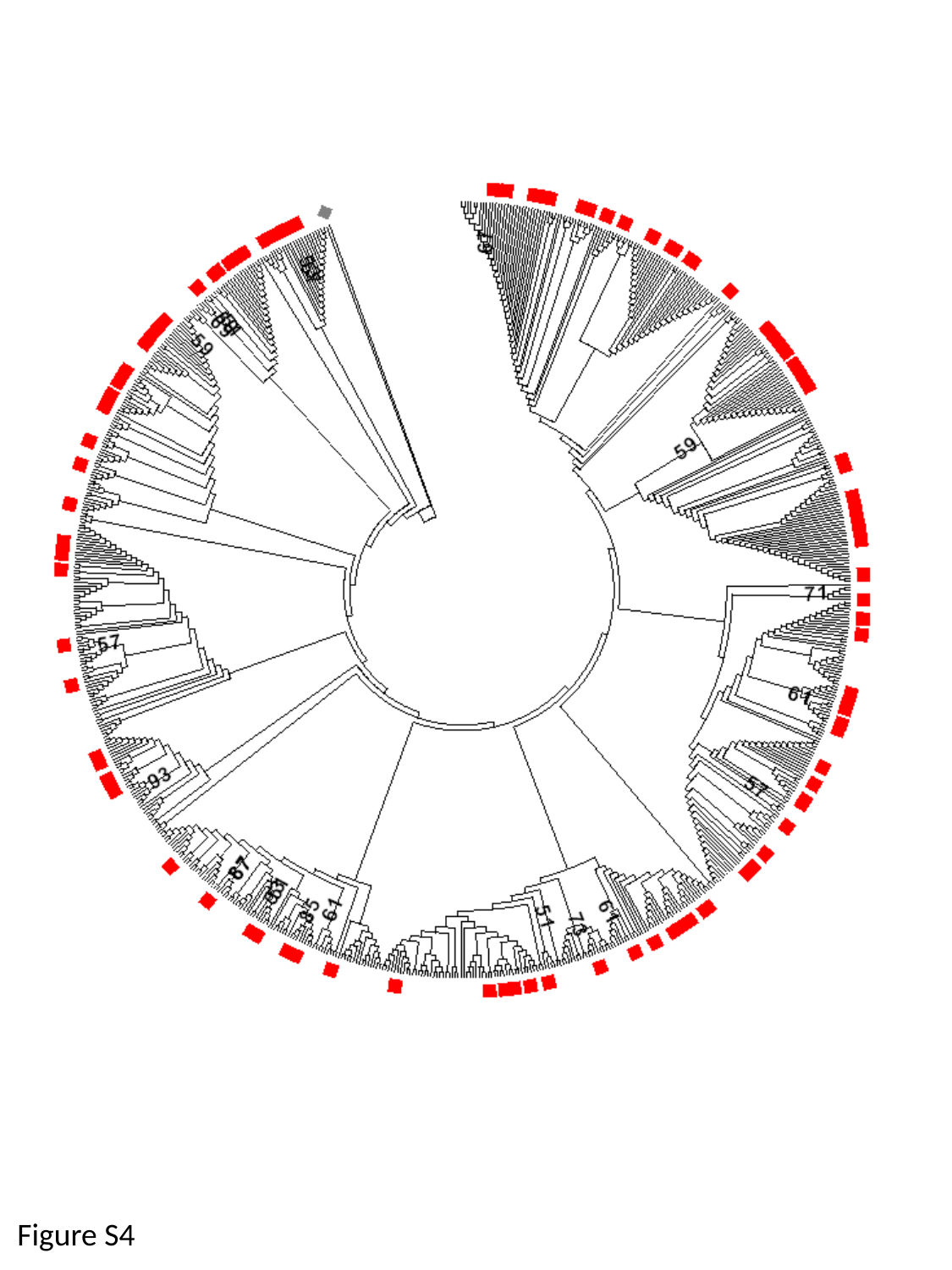

Figure S4

## Slide 5
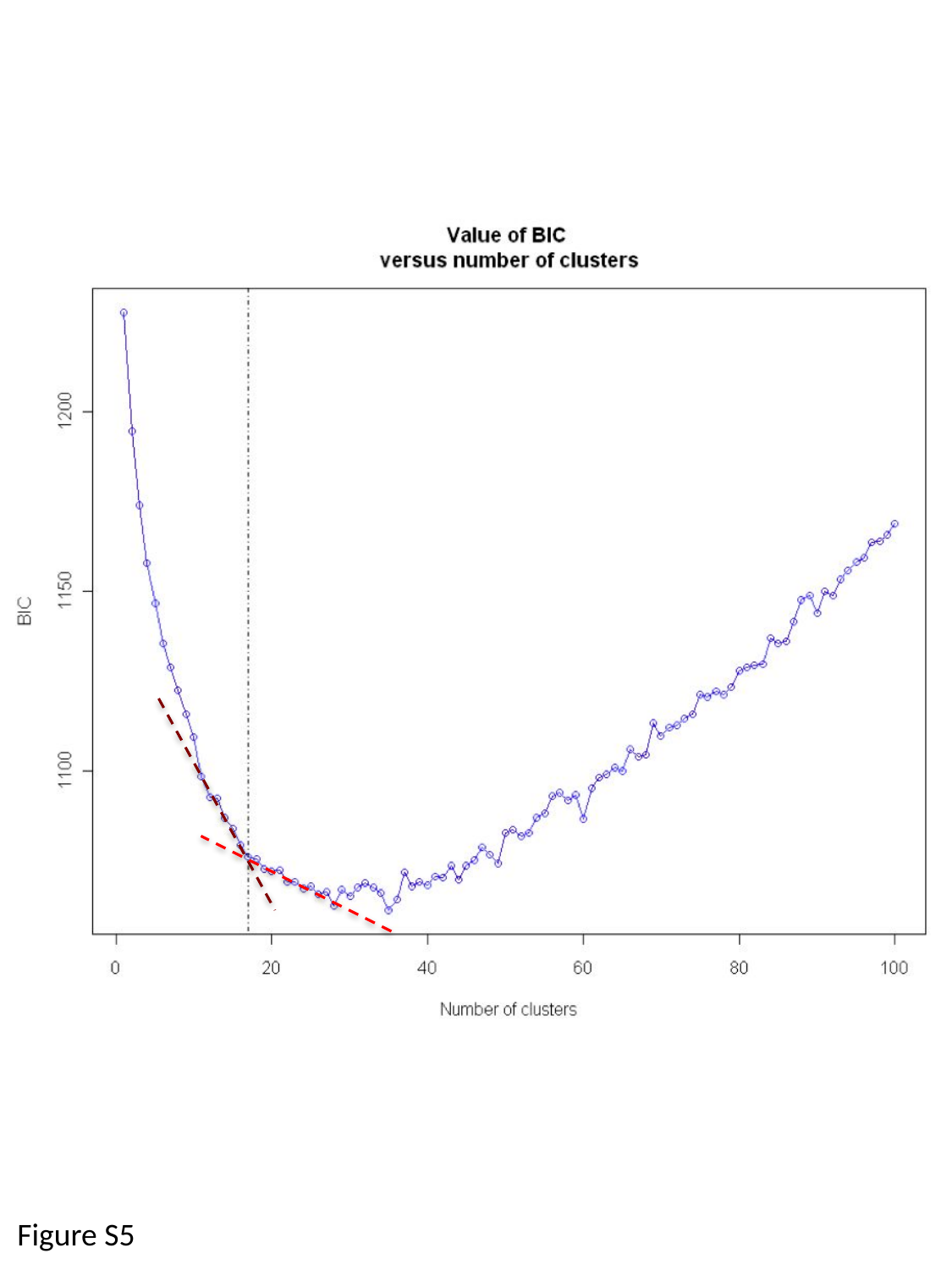

Figure S5

Supplement: Additional file 1 — Fig S1, Fig S2, Fig S3, Fig S4, Fig S5. Figure S1. Phylogenetic tree of the Mhcclass I sequences we identified during characterization. The tree was constructed using Neighbour-Joining method, Jukes-Cantor model and rooted with a Scarlet Rosefinch (Carpodacus erythrinus) Mhc class I exon 3 sequence [Genbank: FJ392790]. The interior branch numbers refer to bootstrap values with 2000 replications. Blue squares indicate sequences retrieved from cDNA. Three sequences adjacent to the Scarlet Rosefinch Mhc allele are the pseudogene alleles and are indicated with the letter ‘P’. Figure S2. Variation in supertype number per individual with increasing read number. Figure S3. Aminoacid sequences of a representative set of great tit Mhc class I alleles (exon 3). The first set of alleles (Pama-U*II001-II758) are members of Group 2; the second set of alleles (Pama-U*I17-I60) are members of Group 1; and the third set of alleles (Pama-U*P15-P34) are members of the pseudogene. Identity with Pama-U*II001 is indicated with dots, differences are shown by letter substitutions, and gaps are shown by dashes. Stop codons are shown by stars. Chicken antigen binding sites (ABS) are shaded with blue, while Group 2 positively selected sites (PSS) are indicated with ‘+’. Pama-U*II758 is one if the three non-functional alleles within Group 2. Figure S4. Phylogenetic tree of functional Group 2 sequences. The tree was constructed using Neighbour-Joining method, Jukes-Cantor model and rooted with a chicken (Gallus gallus) Mhc class I sequence [Genbank: AY234770]. The reliability of the branches was tested with 1000 bootstrap replicates. Bootstrap supports for major clades are indicated with numbers. The alleles that were present in more than 20 individuals are marked in red. The chicken Mhc allele is marked in grey. Figure S5. Graph of BIC values for increasing number of clusters. The optimal number of supertypes was identified as 17 (marked with a vertical, black dashed-line) as it indicated the [file 1471-2148-12-68-S1.pptx]
